# Death from Hypodermic Injection

**Published:** 1869-06

**Authors:** 


Death from Hypodermic Injection.—Lantesson reports that he saw a child die in a few moments with convulsions, after he had injected several drops of liquor ferri sesquichlor. for nævus maternus. Dissection revealed large coagula in the roots of the great veins at the heart and in the right auricle and ventricle. He supposes that a vein of some size was wounded, and that the astringent thus got into the general circulation, coagulated the blood, and finally produced paralysis of the heart. He recommends that the flow of blood into neighboring venus plexuses should be prevented by pressure when we perform this operation.—*St. Louis Med. Reporter.*

